# Two pediatric type II achalasia cases complicated by candidal and eosinophilic esophagitis: Successful peroral endoscopic myotomy treatment

**DOI:** 10.1002/jpr3.70260

**Published:** 2026-10-15

**Authors:** Fabio Rotondo, Antonio Marseglia, Anna Locatelli, Dalila Tedeschi, Marco Gentile, Francesco Perri, Maria Rosa Pastore, Massimo Pettoello Mantovani

**Affiliations:** ^1^ Department of Pediatrics Fondazione IRCCS “Casa Sollievo della Sofferenza” San Giovanni Rotondo Italy; ^2^ Department of Pediatrics University of Foggia Foggia Italy; ^3^ Departmend of Gastroenterology Fondazione IRCCS “Casa Sollievo della Sofferenza” San Giovanni Rotondo Italy

**Keywords:** esophageal motility disorder, high‐resolution manometry, minimally invasive endoscopy, mucosal esophageal disease

## Abstract

Achalasia is a rare esophageal motility disorder in children, often leading to delayed diagnosis. We report the first two pediatric cases treated with peroral endoscopic myotomy (POEM) at our center, both classified as type II achalasia and complicated by mucosal disease. A 15‐year‐old girl presented with progressive dysphagia and weight loss, diagnosed with type II achalasia and Candida esophagitis. A 13‐year‐old boy exhibited similar symptoms with a 27‐kg weight loss; histology revealed concurrent eosinophilic esophagitis. Both underwent successful POEM. These cases illustrate the clinical heterogeneity of pediatric achalasia and emphasize the importance of recognizing concomitant mucosal conditions that may delay diagnosis. POEM appears to be a safe and effective, minimally invasive treatment in the pediatric population, even in complex scenarios.

## Abstract (highlights)

**What Is Known:**

- Pediatric achalasia is rare and frequently diagnosed late, often resulting in severe weight loss.
- High‐resolution manometry is essential for diagnosis and subtype classification.
- Peroral endoscopic myotomy (POEM) is an effective minimally invasive treatment for achalasia, with increasing pediatric evidence.

**What Is New:**

- Concomitant esophageal mucosal diseases may mask achalasia and contribute to significant diagnostic delay in children.
- Achalasia can coexist with candidal or eosinophilic esophagitis, requiring targeted pre‐procedural management.
- POEM remains safe and effective in pediatric achalasia despite associated mucosal disease, allowing rapid nutritional recovery.

## 1 INTRODUCTION

Achalasia is a rare primary esophageal motility disorder characterized by impaired relaxation of the lower esophageal sphincter (LES) and loss of peristalsis, resulting from degeneration of inhibitory neurons in the myenteric plexus.^1^, ^2^ This leads to functional obstruction at the gastroesophageal junction, causing dysphagia, regurgitation, chest pain, and weight loss. High‐resolution manometry (HRM), interpreted according to the Chicago Classification v4.0, is the diagnostic gold standard and allows subtyping into types I, II, or III, with type II showing the best response to therapy.^3^, ^4^

Pediatric achalasia is exceptionally uncommon, with an estimated annual incidence of 0.1–0.18 per 100,000 children.^5^, ^6^ Symptoms often mimic more common conditions such as gastroesophageal reflux or functional dysphagia, leading to delayed recognition.^7^, ^8^ Traditional treatment options include pneumatic dilation (PD), botulinum toxin injection, and laparoscopic Heller myotomy (LHM) with partial fundoplication, but all target symptom control rather than disease reversal.^9^, ^10^, ^11^

Peroral endoscopic myotomy (POEM), first introduced by Inoue et al. in 2010, has emerged as a minimally invasive endoscopic alternative achieving comparable or superior success rates to LHM in adults.^12^ In the pediatric population, recent systematic reviews and pooled analyses report technical and clinical success exceeding 90%.^13^, ^14^, ^15^

Concomitant mucosal diseases such as infectious esophagitis or eosinophilic esophagitis (EoE) may further complicate the clinical picture. An association between EoE and achalasia has been increasingly recognized, with studies reporting a prevalence of achalasia and obstructive motor disorders in up to 15% of patients with EoE.^16^, ^17^ Eosinophilic infiltration may contribute to neuronal degeneration and esophageal dysmotility.^18^ Similarly, esophageal candidiasis has been described as a complication of achalasia, potentially masking the underlying motility disorder and delaying diagnosis.^19^ The presence of these conditions may temporarily shift management toward antifungal or anti‑eosinophilic therapy, postponing definitive treatment for the underlying motility disorder. We report the first two pediatric patients treated with POEM at our institution, both presenting with type II achalasia complicated by either Candida esophagitis or EoE. These cases illustrate how mucosal comorbidities can modify symptom presentation and delay diagnosis, while demonstrating that POEM remains a safe and effective treatment even in such complex settings.

## 2 METHODS

### 2.1 Ethics statement

Written informed consent was obtained from the patients’ legal guardians for publication of this case report and any accompanying images.

This case report was reviewed and deemed exempt by the Institutional Review Board (IRB) of IRCCS “Casa Sollievo della Sofferenza,” as it is a retrospective description of two clinical cases and does not meet the definition of human subjects research under applicable regulations. All patient identifiers have been removed to ensure anonymity.

### 2.2 Case selection and diagnostic work‐up

This was a retrospective review of two pediatric patients diagnosed with type II achalasia who underwent POEM at the Pediatric Unit of IRCCS Casa Sollievo della Sofferenza, Italy, between May 2022 and March 2023. The first case was managed between May and October 2022, and the second between September 2022 and March 2023, including diagnostic work‐up, intervention, and clinical follow‐up. Clinical presentation, anthropometric data, and symptom duration were recorded.

The diagnostic work‐up included barium esophagram, with a single bolus of low‑density barium (100–200 mL) to assess esophageal diameter, column height, and emptying, and upper gastrointestinal endoscopy with biopsies. HRM was performed with a solid‑state catheter; studies were interpreted according to the Chicago Classification v4.0. Achalasia subtypes were defined as follows: type II was characterized by absence of peristalsis and pan‑esophageal pressurization in at least 20% of swallows. The following normal reference values were used (based on institutional standards and validated normative data):

- integrated relaxation pressure (IRP) <15 mmHg (as per Chicago Classification v4.0);
- LES basal pressure 13–43 mmHg;
- upper esophageal sphincter (UES) basal pressure 34–104 mmHg.

In the second case, the HRM catheter could not traverse the esophagogastric junction despite multiple attempts; diagnosis was therefore established by integrating manometric findings (absent peristalsis) with timed barium esophagogram and endoscopic features.

POEM was performed under general anesthesia with a standard anterior approach as described by Inoue et al.^12^ A mucosal incision was made on the anterior esophageal wall approximately 28–32 cm from the incisors, followed by submucosal tunneling across the esophagogastric junction. A selective circular myotomy was extended 1 cm onto the gastric cardia. The mucosal entry was closed with endoclips.

Post‐operative management included at least 24 h of fasting, prophylactic antibiotics, and contrast study to exclude leaks. Oral intake was gradually reintroduced starting with liquids and advancing to semisolids. All patients received proton pump inhibitor (PPI) therapy for at least 1 month after the procedure.

Clinical and nutritional follow‐up was conducted at 1 and 6 months, with assessment of symptoms (Eckardt score), weight, and dietary tolerance. Endoscopic and manometric controls were planned at 6–12 months.

## 3 RESULTS

### 3.1 Case 1

A 15‐year‐old female presented with progressive dysphagia to solids and liquids, nocturnal cough, and a 30‑kg weight loss over 1 year. Symptoms had started in October–November 2021; the diagnosis of achalasia was established in May 2022, with a diagnostic delay of approximately 7 months. Initial investigations included transnasal fiberoptic laryngoscopy (normal, with findings suggestive of gastroesophageal reflux). Timed barium esophagram (100 mL low‐density barium) showed at 0 min a maximal esophageal diameter of 58 mm and barium column height of 188 mm; at 1 min, diameter of 55 mm and column height of 182 mm, findings consistent with achalasia (Figure 1). Esophagogastroduodenoscopy (EGDS) revealed white plaques consistent with Candida esophagitis; biopsies excluded eosinophilic infiltration. The presence of Candida esophagitis initially diverted diagnostic attention toward an infectious etiology, contributing to the delay in diagnosing achalasia. HRM (interpreted according to Chicago Classification v4.0) showed absent peristalsis, pan‑esophageal pressurization, IRP of 39 mmHg (normal <15 mmHg), and elevated LES basal pressure (48 mmHg; normal 13–43 mmHg). Type II achalasia was diagnosed.

**Figure 1:**
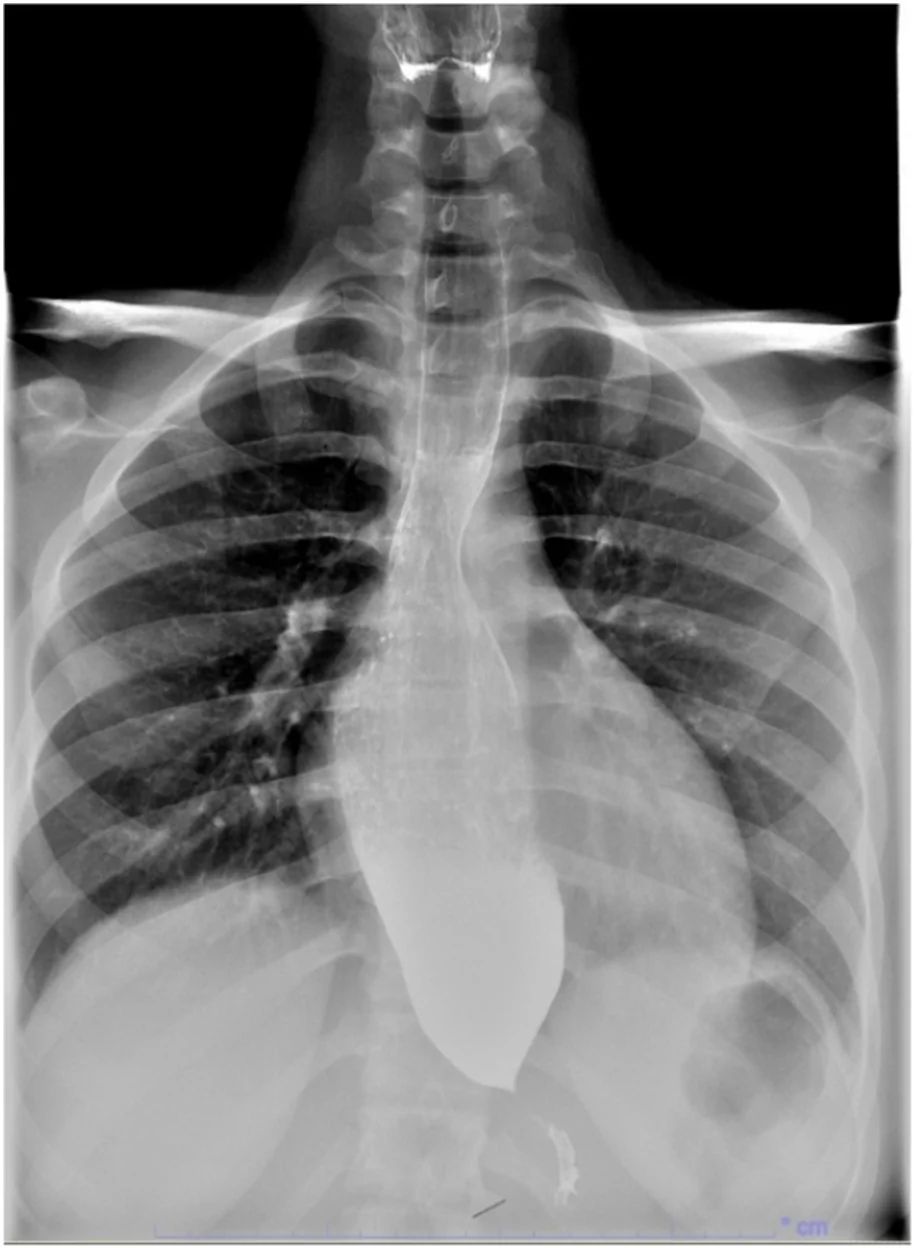
Case 1: Barium esophagram. Tight lower esophageal sphincter stenosis and esophageal dilation after ingestion of 100 mL of low‐density barium, compatible with type II achalasia.

She received intravenous fluconazole, parenteral nutrition, and PPI as bridge therapy. Antifungal treatment and nutritional support were administered prior to intervention, resulting in a planned delay of the procedure until resolution of the active mucosal infection.

POEM was performed using an anterior approach with selective circular myotomy extending 1 cm onto the gastric side. The mucosal entry was closed with endoclips. A contrast study the following day showed no leak. Diet was gradually advanced from liquids to semisolids within 48 h, and she was discharged on PPI therapy.

At 6‐month follow‑up, she remained asymptomatic with a 12‐kg weight regain.

### 3.2 Case 2

A 13‐year‐old male presented with 1 year of progressive dysphagia from solids to liquids, regurgitation, vomiting, and a 27‑kg weight loss. Symptoms had begun around September 2021 and the diagnosis of achalasia was confirmed at our center in September 2022, with an overall diagnostic delay of approximately 12 months.

A barium esophagogram raised suspicion for esophageal achalasia. EGDS performed at another center suggested achalasia, showing a dilated esophagus with a tight, rosette‐like cardia and features of EoE, confirmed histologically (>15 eosinophils/high‐power field [HPF]).

HRM showed absent peristalsis; the catheter could not traverse the esophagogastric junction despite multiple attempts, precluding calculation of IRP and LES relaxation. Despite treatment with budesonide (2 mg/day), lansoprazole (30 mg/day), and an elimination diet for several months, symptoms persisted, delaying definitive treatment for the motility disorder.

At our institution, timed barium esophagram (200 mL low‐density barium) confirmed a tight narrowing at the LES consistent with achalasia (Figure 2). HRM showed absent peristalsis and the catheter could not traverse the esophagogastric junction again; based on the combination of absent peristalsis, barium esophagogram findings, and endoscopic features, a diagnosis of type II achalasia was made.

**Figure 2:**
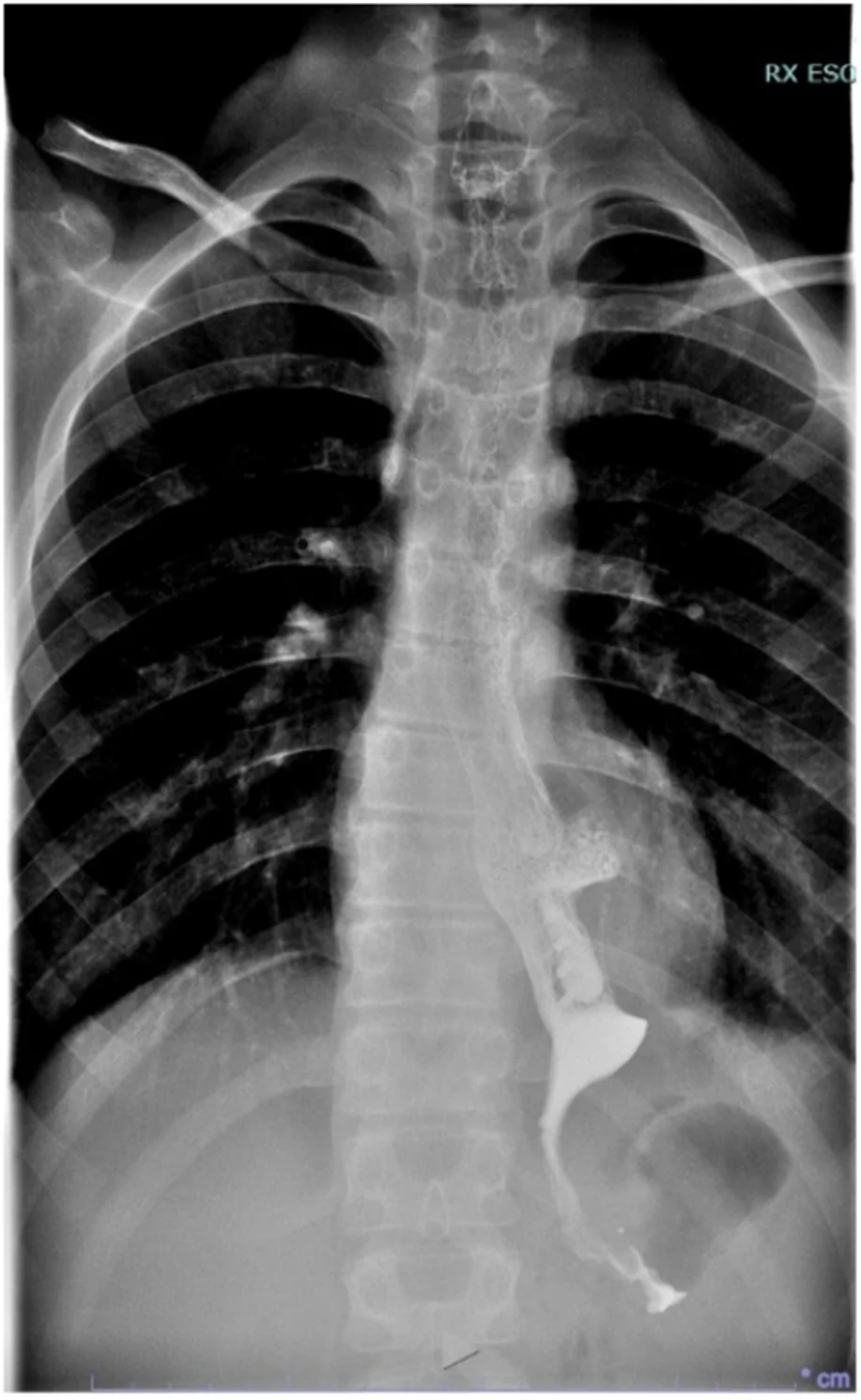
Case 2: Barium esophagram. Tight lower esophageal sphincter stenosis and esophageal dilation after ingestion of 200 mL of low‐density barium, compatible with type II achalasia.

POEM was performed using a standard anterior approach with selective circular myotomy and extending 1 cm onto the gastric side; the mucosal entry was closed with endoclips. A post‐operative contrast study showed no leak. Oral intake resumed within 48 h, with a slow advancement from liquids to semisolids. At the time of discharge, the patient tolerated liquids and semisolids well, with persistent mild dysphagia for solids.

At 3 months, he had normal swallowing, no reflux symptoms, and a 13‑kg weight gain.

At 6‐month endoscopy, the esophagus appeared mildly dilated with normal mucosa; biopsies showed no eosinophilic infiltration (<15/HPF), with the patient continuing PPI therapy. Thus, histological remission of EoE was achieved after POEM under ongoing PPI treatment.

Both patients remained asymptomatic at medium‐term follow‐up. No adverse events, leaks, or significant reflux symptoms were observed. POEM achieved complete clinical success and normalization of oral intake in both cases.

## 4 DISCUSSION

Achalasia is rare in childhood and often presents with variable and nonspecific symptoms overlapping with more common gastrointestinal disorders, contributing to delayed diagnosis.^5^, ^6^, ^7^, ^8^ Early identification is crucial to avoid malnutrition, esophageal dilation, and psychosocial consequences.^5^

In our two cases, both adolescents had a prolonged diagnostic delay (7 and 12 months, respectively). Notably, in both instances, concomitant mucosal diseases—Candida esophagitis in Case 1 and EoE in Case 2—initially diverted clinical attention toward infectious or allergic/inflammatory etiologies, thereby postponing the correct diagnosis of achalasia.

The association between achalasia and EoE is increasingly recognized. Studies report that up to 15% of patients with EoE may have achalasia or other obstructive motor disorders.^16^, ^17^ Eosinophilic infiltration may induce neuronal dysfunction and esophageal remodeling, potentially contributing to the pathogenesis of achalasia.^18^ Similarly, esophageal candidiasis is a well‑documented complication of achalasia due to stasis; however it may also mask the underlying motility disorder.^19^ In our first case, the presence of Candida esophagitis led to initial management focused on antifungal therapy, delaying the diagnosis of achalasia by several months. In the second case, despite 6 months of targeted anti‑eosinophilic therapy (topical budesonide, PPI, and elimination diet), symptoms persisted, and only after withdrawal of these treatments and completion of the diagnostic work‑up was achalasia confirmed.

HRM, interpreted according to the Chicago Classification v4.0, is the gold standard for diagnosis and subtype classification.^3^, ^4^, ^20^ In our second case, the HRM catheter could not traverse the esophagogastric junction due to severe stenosis—a situation that might be misinterpreted as technical failure. However, the combination of absent peristalsis on HRM, typical findings on timed barium esophagogram (tight narrowing at the LES with delayed emptying), and endoscopic features (dilated esophagus with a tight, rosette‑like cardia) allowed a confident diagnosis of type II achalasia. This case also illustrates that when HRM is incomplete, complementary diagnostic tools are essential.

Treatment options include PD, botulinum toxin injection, and LHM, and POEM.^9^, ^10^, ^11^ PD is minimally invasive but carries a risk of perforation (approximately 2%–5%) and symptom recurrence is common, often requiring repeated procedures.^9^ LHM offers durable results but requires surgical access; most centers perform a partial fundoplication (e.g., Dor) to reduce the risk of post‐operative gastroesophageal reflux.^10^, ^11^ Botulinum toxin provides only temporary relief and is reserved for patients unfit for definitive intervention.^7^ POEM has emerged as a highly effective endoscopic alternative, achieving comparable or superior success rates to LHM in adults, with the advantages of minimal invasiveness and rapid recovery.^12^, ^21^, ^22^ In children, recent systematic reviews and meta‑analyses report technical and clinical success rates exceeding 90%.^13^, ^14^, ^15^ Our two cases further support the safety and efficacy of POEM in the pediatric population, even in the presence of complicating mucosal diseases. Both patients achieved complete symptom resolution and significant nutritional recovery without adverse events or post‐operative leaks. The coexistence of mucosal conditions did not compromise the technical success or safety of POEM. In Case 1, the Candida infection was treated pre‑operatively, and POEM was performed after resolution of active esophagitis. In Case 2, POEM was carried out despite ongoing EoE; interestingly, at 6‐month follow‑up endoscopy, esophageal biopsies showed no eosinophilic infiltration (<15/HPF) while the patient was on maintenance PPI therapy. This suggests that relief of stasis after myotomy may contribute to the improvement of eosinophilic inflammation although the role of PPI cannot be excluded. This observation aligns with the concept that mechanical obstruction can drive eosinophilic infiltration and that relieving the obstruction may improve histological outcomes.^18^

Limitations include the small sample size and single center study design. Nonetheless, as the first pediatric POEMs at our institution, these cases demonstrate feasibility, safety, and clinically meaningful improvement, consistent with published pediatric series.^13^, ^14^, ^15^

## 5 CONCLUSION

POEM offers an excellent therapeutic alternative for pediatric achalasia, combining high efficacy with minimal invasiveness, even in the presence of complicating esophageal mucosal disease. These cases highlight the feasibility of POEM in complex pediatric patients and support its expanding role as a first‐line therapeutic option in specialized centers. Further multicenter prospective studies are warranted to validate long‐term outcomes and refine patient selection in this population.

## CONFLICT OF INTEREST STATEMENT

The authors declare no conflicts of interest.

## Data Availability

The data that support the findings of this study are available from the corresponding author upon reasonable request.
